# Human Infections with *Borrelia miyamotoi*, Japan

**DOI:** 10.3201/eid2008.131761

**Published:** 2014-08

**Authors:** Kozue Sato, Ai Takano, Satoru Konnai, Minoru Nakao, Takuya Ito, Kojiro Koyama, Minoru Kaneko, Makoto Ohnishi, Hiroki Kawabata

**Affiliations:** National Institute of Infectious Diseases, Tokyo, Japan (K. Sato, M. Ohnishi, H. Kawabata);; Yamaguchi University, Yamaguchi, Japan (A. Takano);; Hokkaido University, Sapporo, Japan (S. Konnai);; Asahikawa Medical College, Asahikawa, Japan (M. Nakao);; Hokkaido Institute of Public Health, Sapparo (T. Ito);; Oumu National Health Insurance Hospital, Oumu, Japan (K. Koyama);; Kamifurano Hospital, Kamifurano, Japan (M. Kaneko, Japan);; Gifu University, Gifu, Japan (H. Kawabata)

**Keywords:** Borrelia miyamotoi, bacteria, relapsing fever, Lyme disease, Ixodes persulcatus, ticks, vector-borne infections, Japan

## Abstract

We confirmed infection of 2 patients with *Borrelia miyamotoi* in Japan by retrospective surveillance of Lyme disease patients and detection of *B. miyamotoi* DNA in serum samples. One patient also showed seroconversion for antibody against recombinant glycerophosphodiester phosphodiesterase of *B. miyamotoi*. Indigenous relapsing fever should be considered a health concern in Japan.

*Borrelia miyamotoi*, which is genetically grouped with relapsing fever borreliae, was recently identified as a human pathogen in Russia (*1*), the United States (*2*–*4*), and Europe (*5*). Ticks of the *Ixodes persulcatus* species complex are transmission vectors. Pathogenic borreliae were discovered in *I. persulcatus* ticks in Japan (*6*).

In areas of Japan to which Lyme disease is endemic, wild rodents have been found to be infected with *B. miyamotoi* (*7*), although no human infections have been confirmed. *B. miyamotoi* isolates from Japan are potential human pathogens because they form a monophyletic lineage with isolates from patients in Russia (*1*). We conducted a retrospective investigation to identify occult cases of human infections with *B. miyamotoi* in Japan.

## The Study

A total of 615 serum samples were obtained from 408 persons in Japan who had confirmed Lyme disease or unconfirmed, clinically suspected Lyme disease and used to detect *B. miyamotoi* DNA. The serum archive was established during 2008–2013 at the National Institute of Infectious Diseases (Tokyo, Japan). Use of human samples was approved by the ethical committee of the National Institute of Infectious Diseases for medical research with humans (approval no. 360; July 2, 2012).

All serum samples were centrifuged (15,000 × *g* for 10 min), and sediments were used for DNA extraction. DNA extraction was performed by using the DNeasy Blood and Tissue Kit (QIAGEN, Hilden, Germany) according to the manufacturer’s instructions with minor modification (the extraction column was incubated for 10 min at 70°C before DNA was collected).

For detection of *B. miyamotoi* DNA, real-time PCR was performed with primers and probes described by Barbour et al. (*8*). The reaction was performed in a 25-μL volume in single tubes with 1 µmol/L of each primer and 0.25 µmol/L of each probe. The PCR was conducted on a 7000 Real Time PCR Apparatus (Applied Biosystems, Foster City, CA, USA), and conditions were 42 cycles at 95°C for 5 s and 60°C for 31 s. For confirmation of positive samples, a *flaB *gene nested PCR and sequencing of amplicons were performed as described (*9*).

The number of copies of DNA in patient serum samples was estimated by quantitative PCR (qPCR). Plasmid pBMrrs1 that contained part of the 16S rRNA gene for *B. miyamotoi* strain HT31 was prepared as described (*9*) and was used as a quantitative control.

A recombinant *B. miyamotoi* glycerophosphoryl diester phosphodiesterase (GlpQ) was used for serologic testing. The GlpQ gene (*glpQ)* of *B. miyamotoi* strain HT31 was cloned into plasmid vector pET-19b (Merck KGaA, Darmstadt, Germany). His-tagged GlpQ was subsequently expressed in *Escherichia coli* Rossetta strain (Merck KGaA) by using MagicMedia (Life Technologies, Carlsbad, CA, USA). Western blotting for detection of antibodies against GlpQ in patient serum was performed as described (*10*). Our retrospective investigation identified 2 cases of *B. miyamotoi* infection.

Case-patient 1 was a previously healthy, 72-year-old woman who lived in Hokkaido, Japan. The patient reported no history of foreign travel. Myalgia and anorexia developed on July 23, 2011, and she was hospitalized on July 25, at which time she had a fever of 39°C. Physical examination showed erythema migrans, and the patient confirmed that she had been bitten by a tick 10 days earlier.

Laboratory tests on July 25 showed increased levels of C-reactive protein (44 mg/L), alanine aminotransferase (94 IU/mL), and aspartate aminotransferase (90 IU/ml). The procalcitonin level was ≥0.05 ng/mL, as determined by using a PCT-Q Test (Brahms GmbH, Hennigsdorf, Germany). The leukocyte count was 3,900 cells/μL (87% neutrophils), and a left shift was observed. Erythrocyte and platelet counts were within reference ranges. The patient was given a clinical diagnosis of acute Lyme disease because of typical erythema migrans and a history of a tick bite. The patient was treated with minocycline (100 mg/day for 5 days). Symptoms improved rapidly, and she was discharged from the hospital on July 30.

Borrelial DNA was detected in a serum sample obtained on July 25 by 16S rRNA gene–based-real time qPCR and a *flaB* gene–specific nested PCR. Sequencing of a *flaB *PCR amplicon (294 bp) indicated that the infectious borreliae was *B. miyamotoi* (GenBank accession no. AB921566) because the sequence was identical to that of *B. miyamotoi* HT31 (GenBank accession no. D43777). The number of copies of the *Borrelia* genome in serum was estimated to be 7.2 × 10^3^ copies/mL by qPCR.

The level of IgM against GlpQ was increased in a convalescent-phase serum sample obtained on August 10 (Figure, panel A). We also found that antibody levels were increased in convalescent-phase serum by conducting immunoblot analysis with a whole cell lysate of *B. miyamotoi* strain MYK3 (Figure, panel B). 

**Figure:**
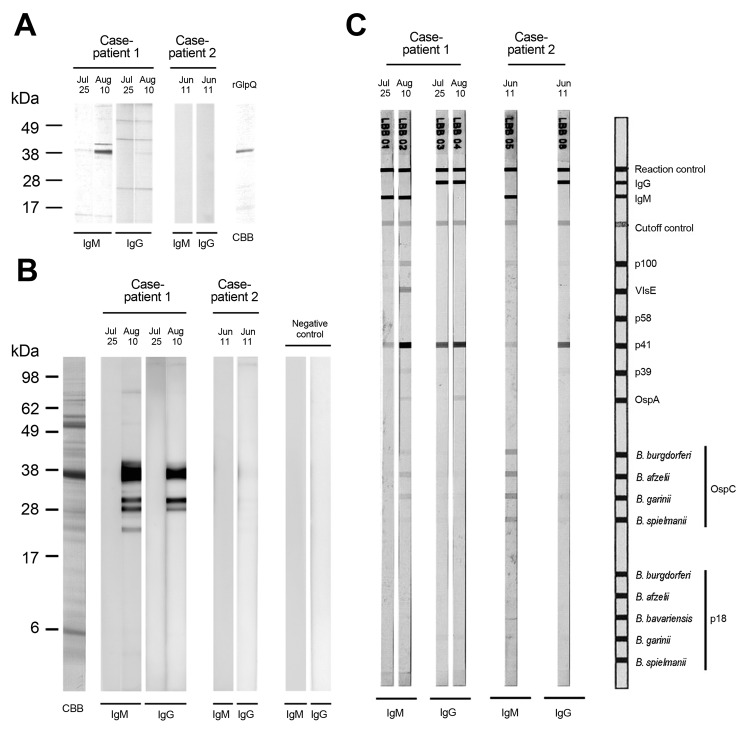
Immunoblot analysis of serum reactivity to antigens of *Borrelia miyamotoi* and Lyme disease borreliae, Japan. Serum samples obtained from 2 patients were examined. For case-patient 1, acute-phase serum obtained on July 25, 2011, and convalescent-phase serum obtained on August 10 were used. For case-patient 2, acute-phase serum obtained on June 11 was used. A) Reactivity to recombinant glycerophosphodiester phosphodiesterase (GlpQ) antigen. Crude rGlpQ were used for immunoblot analysis (*10*). Recombinant GlpQ was separated by electrophoresis on a 5%–20% polyacrylamide gradient gel (Wako Pure Chemical Industries Inc., Osaka, Japan), and antigen was stained with Coomassie brilliant blue. CBB, protein profile. Molecular mass markers are shown on the left. B) Reactivity of patient serum samples to whole cell lysate of *B. miyamotoi* antigens. A low-passage strain of *B. miyamotoi* (strain MYK3) was used for immunoblot analysis (*10*). A negative control was serum obtained from a healthy human (resident of an area to which Lyme disease was not endemic). Molecular mass markers are shown on the left. C) Serodiagnosis of Lyme disease by immunoblot analysis of serum samples from the 2 patients. OspC, outer surface protein C.

Case-patient 2 was a previously healthy 37-year-old man who lived in Hokkaido, Japan. The patient reported no history of foreign travel. The patient was bitten by a tick on May 28, 2013, and was subsequently hospitalized on June 11, at which time he had a fever of 39.8°C and erythema migrans at the site of the tick bite. The patient was given a clinical diagnosis of acute Lyme disease and erythema migrans. The patient was treated with ceftriaxone (1 g/day for 7 days). Symptoms improved rapidly, and treatment was administrated until July 17 in the outpatient setting.

Borrelial DNA was detected from a serum sample obtained on June 11 by qPCR and nested *flaB *PCR. Sequencing of the *flaB *PCR amplicon (294 bp) confirmed *B. miyamotoi* (GenBank accession no. AB921567) infection in the patient because the sequence was identical to *B. miyamotoi* HT31 (GenBank accession no. D43777). The number of copies of the borrelia genome in serum was estimated to 2.8 × 10^4^ copies/mL by qPCR. Antibodies against GlpQ were not detected in serum obtained on June 11 (Figure, panel A).

Serologic analysis with a commercial kit for IgM (RecomLine Borrelia IgG/IgM; Mikrogen, Neuried, Germany) (Figure, panel C) showed that serum from these 2 patients reacted with several antigens of Lyme disease borreliae (Figure, panel C). Convalescent-phase serum from case-patient 1 reacted to P100 from *B. afzelii*, VlsE from various *Borrelia *species, P41 from *B. burgdorferi* sensu stricto, and OspC from *B. afzelii* and *B. garinii*. Acute-phase serum from case-patient 2 reacted with OspC for all *Borrelia* species included in the kit. However, the commercial serologic test used does not provide enough evidence to determine whether these 2 patients were co-infected with Lyme disease borreliae because antigenic difference between *B. miyamotoi* and *B. burgdorferi* sensu lato have not been investigated.

## Conclusions

Platonov et al. (*1*) reported that *I. persulcatus* ticks are a transmission vector for *B. miyamotoi* and Lyme disease borreliae in Russia. This tick species is also ubiquitous in Hokkaido, Japan, and host-seeking behavior of adult ticks is active during spring–late summer. Humans in Hokkaido are bitten most often by *I. persulcatus* ticks, and Lyme disease borrelia is transmitted to humans mainly through the bite of the adult tick (*11*). Although the causative tick species was not identified for the 2 case-patients, circumstantial evidence suggests that *I. persulcatus* ticks are a main transmission vector for *B. miyamotoi* in Hokkaido, as shown in Russia (*1*).

Emerging relapsing fever caused by *B. miyamotoi* has been identified in Russia, North America, and Europe, and *B. miyamotoi*-related meningoencephalitis has been reported in the United States and the Netherlands. Our study indicates that a human health threat from emerging relapsing fever is present in Japan. For risk analysis of this emerging relapsing fever, epidemiologic surveys (e.g., determining infection rates of host-seeking ticks of the *I. persulcatus* species complex in various locations in Japan) and improvement of serologic diagnostic systems (especially early diagnosis) should be considered.
